# Supplementary material for: Synthesis of Soluble High Molar Mass Poly(Phenylene Methylene)-Based Polymers
Source: Polymers (Basel). 2024 Apr 2;16(7):967. doi: 10.3390/polym16070967 (PMC11014094; doi:10.3390/polym16070967)
Supplement: Supplementary file 1 [file polymers-16-00967-s001.zip › polymers-2931622-supplementary.pdf]

polymers

Synthesis of Soluble High Molar Mass Poly(Phenylene Methylene)-Based Polymers

Marco F. D'Elia [1,*], Yingying Yu [1], Melvin Renggli [1], Madeleine A. Ehweiner [2], Carina Vidovic [2], Nadia C. Mösch-Zanetti [2], Markus Niederberger [1] and Walter Caseri [1,*]

[1] Laboratory for Multifunctional Materials, Department of Materials, ETH Zürich, 8093 Zürich, Switzerland; yingyu@student.ethz.ch (Y.Y.); rengglme@student.ethz.ch (M.R.); markus.niederberger@mat.ethz.ch (M.N.)
[2] Institut für Chemie/Bereich Anorganische Chemie, Universitaet Graz, Schubertstraße 1/3, 8010 Graz, Austria; madeleine.ehweiner@uni-graz.at (M.A.E.); vidovic.carina@web.de (C.V.); nadia.moesch@uni-graz.at (N.C.M.-Z.)
* Correspondence: marco.delia@mat.ethz.ch (M.F.D.); walter.caseri@mat.ethz.ch (W.C.)

Figure S1. ^{13}C NMR spectra of PPM homopolymers synthesized by polymerization of benzyl chloride with the catalysts indicated in the diagram.

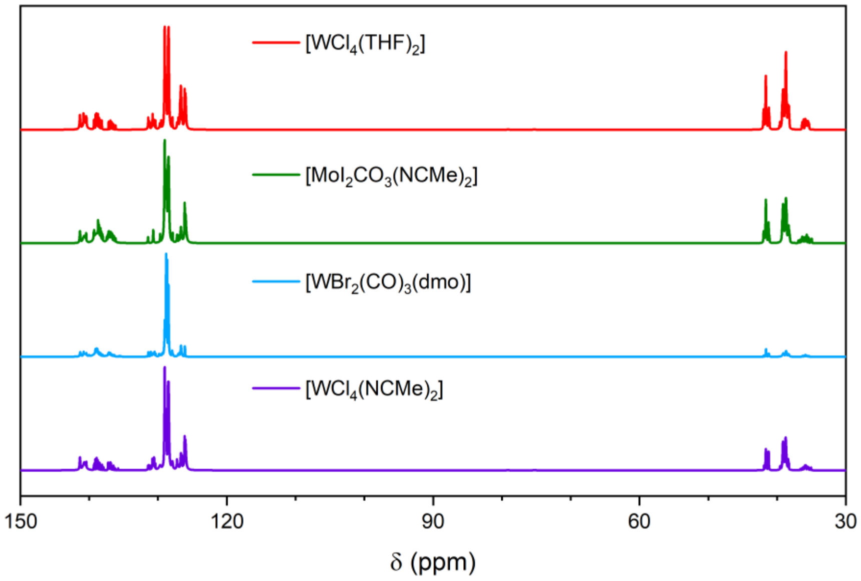

Figure S2. ^{1}H NMR spectra corresponding to the above ^{13}C NMR spectra.

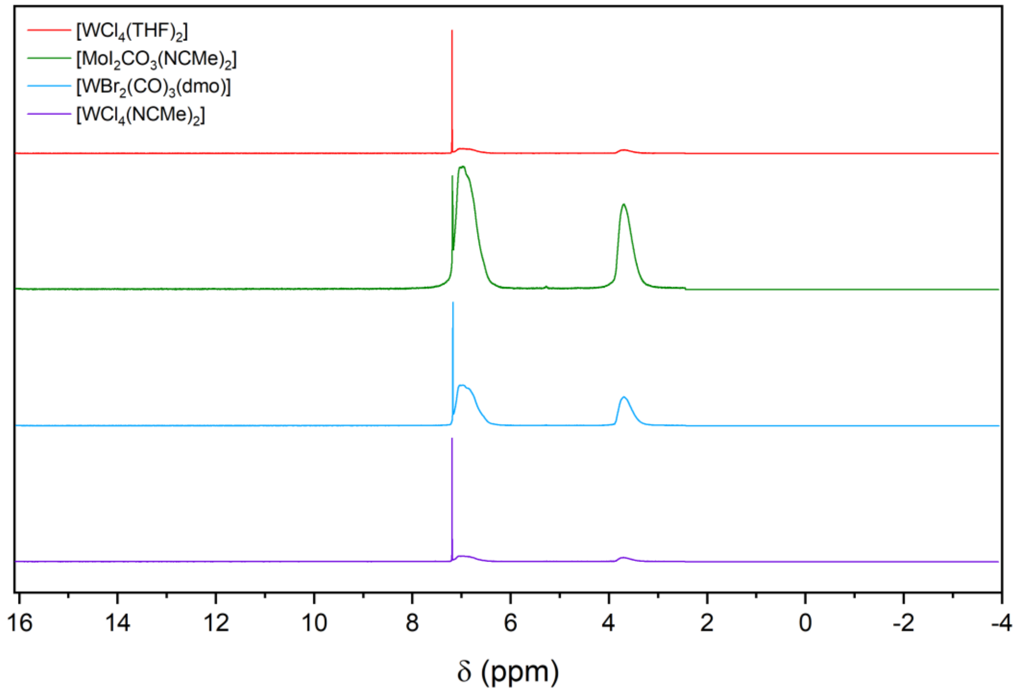

Figure S3. ^{1}H NMR spectra of the PPM copolymer containing BCMD.

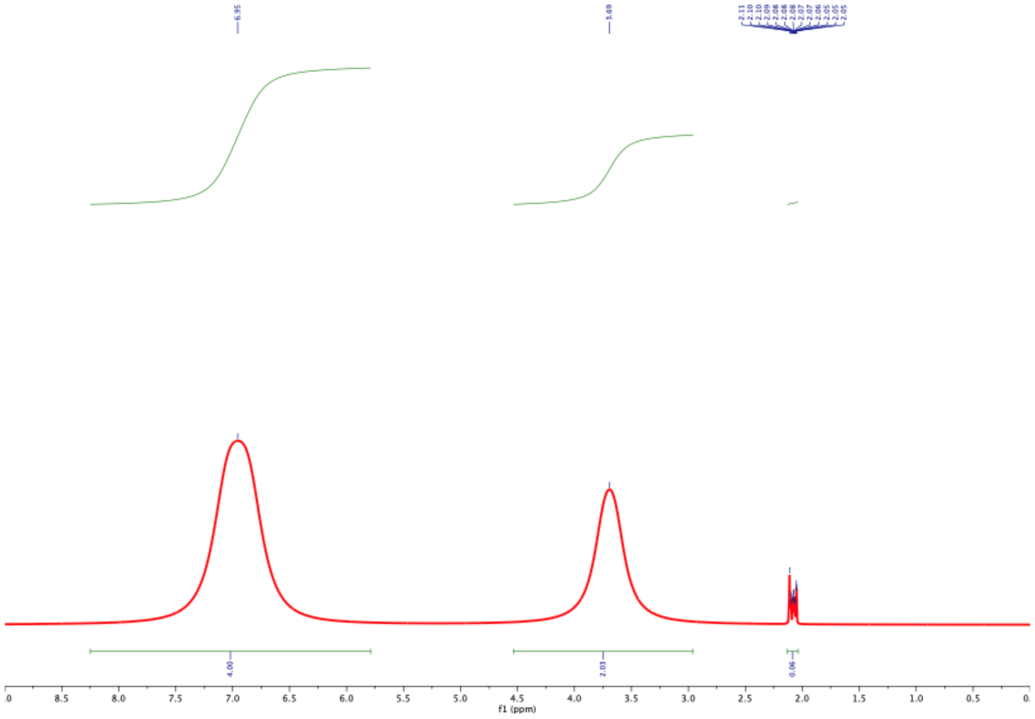

Figure S4. Photoluminescence emission spectra of PPM obtained by polymerization of benzyl chloride with $SnCl_4$ as catalyst.

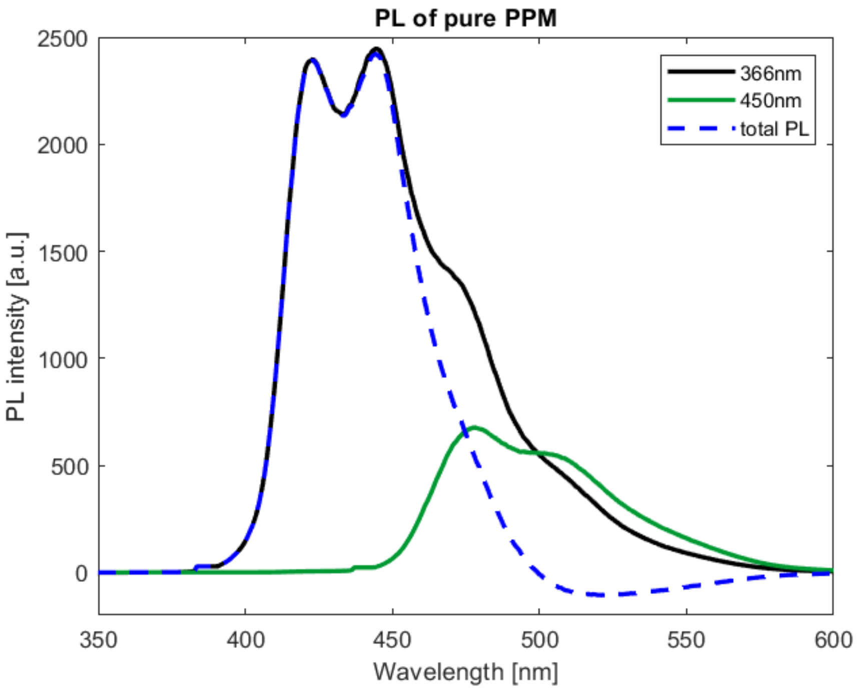

Figure S5. Photoluminescence excitation spectra of PPM obtained by polymerization of benzyl chloride with $SnCl_4$ as catalyst.

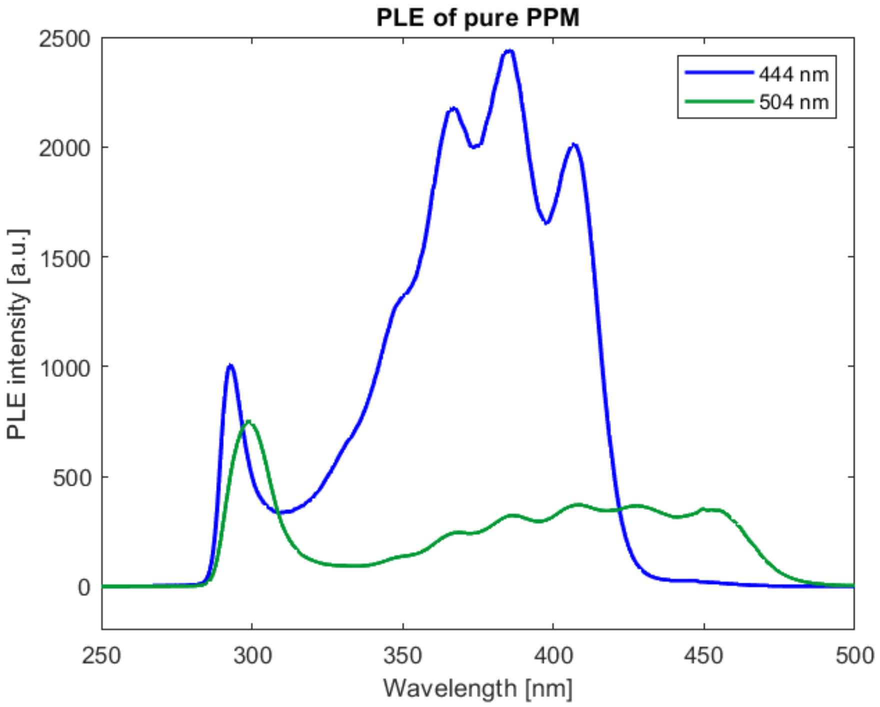

Synthesis of PPM and PPM based polymer catalyzed by Bi(OSO2CF3)3

A quantity of 14.7 g (0.1 mol) of benzyl chloride was combined with 0.47 % mol/mol 3.6-bis (chloromethyl) durene, BCMD (0.1 g). The reaction was carried out using $Bi(OSO_2CF_3)_3$ as catalyst (1 % mol/mol with respect to the overall number of moles), keeping a constant nitrogen flow (20 mL min−1). The polymerization reaction was initiated at 80 ◦C. To overcome mixing issues arising from the increase of viscosity of the reaction mixture, the temperature was raised up during the course of the polymerization as following: 120 ◦C for 4 h, 160 ◦C 8 h and finally 180 C 2 h. The obtained polymers were purified by dissolution in chloroform (1.45 mL of solvent for 1 g of initially used benzyl chloride) and precipitation in methanol with a ratio 20 mL of methanol for 1 mL of chloroform. Same procedure was performed also for the homopolymerization of benzyl chloride.

Catalyst syntheses

All synthetic manipulations were performed under a nitrogen atmosphere using standard Schlenk and glovebox techniques. Solvents were purified via a Pure Solv Solvent Purification System. Chemicals were purchased from commercial sources and used without further purification. $W_2Br_4(CO)_7$ was prepared according to a literature procedure.[1] The NMR spectrum was recorded on a Bruker Avance III 300 MHz spectrometer at ambient temperature. Chemical shifts δ are given in ppm. The multiplicity of peaks is denoted as singlet (s). NMR solvents were stored over molecular sieves. Solid state IR spectra were measured on a Bruker ALPHA ATR-FT-IR spectrometer at a resolution of 2 cm^{-1}. Elemental analysis was performed at the Department of Inorganic Chemistry at the University of Technology in Graz; values are given as percentages.

Synthesis of $[WBr_2(CO)_3(dme)]$: A 250 mL Schlenk flask was charged with $[W_2Br_4(CO)_7]$ (8.00 g, 18.11 mmol) and 80 mL of dme. The resulting orange-red mixture was allowed to stir for 1 h before black solids were removed by filtration. Slow reduction of the volume of the filtrate under reduced pressure initiated the formation of deep red crystals on the flask wall. Evaporation to dryness gave $[WBr_2(CO)_3(dme)]$ (8.85 g, 94%). The compound is air-sensitive and decomposes in chlorinated hydrocarbons upon prolonged exposure. he molecular structure as determined by single crystal X-ray diffraction analysis has been previously published as a CSD Communication.[2] ^1H NMR (CD_2Cl_2, 300 MHz): δ 4.14 (s, 4H, CH_2), 4.00 (s,

6H, CH_3) ppm. IR (C≡O, cm^{-1}): 2015, 1938, 1879. Analysis calculated for $C_7H_{10}O_5Br_2W$: C, 16.24; H, 1.95. Found: C, 16.05; H, 1.87.

$[MoI_2(CO)_3(NCMe)_2]$,[3] $[WCl_4(NCMe)_2]$,[4] and $[WCl_4(THF)_2]$[5] were synthesized according to the literature. The purity of the compounds was assessed by IR spectroscopy.

References

(1) Peschel, L. M.; Schachner, J. A.; Sala, C. H.; Belaj, F.; Mösch-Zanetti, N. C. An Update on W II and Mo II Carbonyl Precursors and Their Application in the Synthesis of Potentially Bio-Inspired Thiophenolate-Oxazoline Complexes. *Z. anorg. allg. Chem.* **2013**, *639* (8-9), 1559–1567.

(2) Belaj, F.; Vidovic, C.; Mösch-Zanetti, N. *CSD Commun.* **2021** CCDC 2064083

(3) Baker, P. K.; Fraser, S. G.; Keys, E. M. The synthesis and spectral properties of some highly reactive new seven-coordinate molybdenum(II) and tungsten(II) bisacetonitrile dihalogenotricarbonyl complexes. *J. Organomet. Chem.* **1986**, *309* (319-321).

(4) Kolesnichenko, V.; Swenson, D. C.; Messerle, L. Facile Reduction of Tungsten Halides with Nonconventional, Mild Reductants. *Inorg. Chem.* **1998**, *37*, 3257–3262.

(5) Persson, C.; Andersson, C. Reduction of tungsten(VI) and molybdenum(V) by allyltrimethylsilane and cyclopentene. *Inorg. Chim. Acta* **1993**, *203*, 235–238.
